# P-1985. Effect of vaccination on long COVID risk: An analysis of 3.6 million older US adults

**DOI:** 10.1093/ofid/ofae631.2143

**Published:** 2025-01-29

**Authors:** Yasin Abul, Daniel Harris, Preeti Chachlani, Kaley Hayes, Vincent Mor, Stefan Gravenstein

**Affiliations:** Brown University, Providence, Rhode Island; Department of Epidemiology, College of Health Sciences, University of Delaware, Newark, Delaware; Center for Gerontology and Healthcare Research, Brown University, Bellingham, Massachusetts; Brown University, Providence, Rhode Island; Brown University, School of Public Health, Providence, Rhode Island; Brown University, Providence, Rhode Island

## Abstract

**Background:**

Long COVID is a disabling condition that can develop after a SARS-CoV-2 infection. There is limited population-based evidence on the risk of long COVID and the potential protective effects of vaccination, especially among older adults. Our objective was to quantify the association between vaccination status and long COVID risk in older adults using a very large sample of older US adults.

**Methods:**

We conducted a cohort study of Medicare beneficiaries with COVID-19 (index date) between Oct 1, 2021 and Mar 31, 2023. Community living beneficiaries ≥66 years old, enrolled in Medicare fee-for-service (FFS) were included. Over one year of followup, we measured long COVID diagnoses (U09.9) in the Medicare Part A/ B claims. Individuals who died or disenrolled were censored. Baseline demographics, comorbidities, prior COVID-19 diagnoses, and number of vaccines were measured as of the index date. Cox proportional hazards estimated cause-specific relative rates of long COVID and Fine & Gray competing risk regression estimated absolute risk.

**Results:**

We identified 3,588,671 eligible Medicare beneficiaries (mean age=76.3; 58% women; 4.6% Black; 12.2% prior COVID-19 diagnosis). A total of 3.2% of beneficiaries were diagnosed with long COVID over follow-up. The adjusted risk of long COVID was higher among beneficiaries with no prior vaccines (4.3%) and lowest among those with four or more prior vaccines (2.5%). The relative rate of long COVID decreased with each vaccine compared to no vaccination (one vaccine adjusted hazard ratio [aHR] = 0.73, 95% confidence interval [CI] = 0.72-0.74; 2 vaccines aHR=0.72, 95%CI=0.71-0.74; 3 vaccines aHR=0.67, 95%CI=0.66-0.68; ≥4 vaccines aHR=0.60, 95%CI=0.59-0.61).

**Conclusion:**

The risk of long COVID was very low in US Medicare beneficiaries diagnosed with COVID-19, likely due to underreporting and undercoding. Despite low coding rates of long COVID in Medicare claims, a significant dose-response relationship was observed with the number of prior COVID-19 vaccines and reduced risk of long COVID. Efforts to improve the clinical detection and documentation of long COVID in administrative databases will be important for public health surveillance, and vaccinations represent an effective strategy at mitigating long COVID risk in older adults.

**Disclosures:**

Yasin Abul, MD, Moderna: Grant/Research Support|Moderna, Abt, CDC: Grant/Research Support Daniel Harris, PhD, MPH, Sanofi Vaccines: Advisor/Consultant Kaley Hayes, PharmD, PhD, Genentech: Grant/Research Support|GlaxoSmithKline: Grant/Research Support|Sanofi: Grant/Research Support Vincent Mor, Ph.D., naviHealth: Advisor/Consultant Stefan Gravenstein, MD, MPH, CDC: Advisor/Consultant|CDC: Grant/Research Support|Genentech: Advisor/Consultant|Genentech: Grant/Research Support|Genentech: Honoraria|GlaxoSmithKline: Advisor/Consultant|GlaxoSmithKline: Grant/Research Support|GlaxoSmithKline: Honoraria|Janssen: Advisor/Consultant|Janssen: Grant/Research Support|Janssen: Honoraria|Moderna: Advisor/Consultant|Moderna: Grant/Research Support|Moderna: Honoraria|NIH: Grant/Research Support|Pfizer: Advisor/Consultant|Pfizer: Grant/Research Support|Pfizer: Honoraria|Sanofi: Advisor/Consultant|Sanofi: Grant/Research Support|Sanofi: Honoraria|Seqirus: Advisor/Consultant

